# Simultaneous piezoelectric noninvasive detection of multiple vital signs

**DOI:** 10.1038/s41598-019-57326-6

**Published:** 2020-01-15

**Authors:** Areen Allataifeh, Mahmoud Al Ahmad

**Affiliations:** Department of Electrical Engineering, College of Engineering, United Arab Emirates University, Al Ain, 15551 UAE

**Keywords:** Electrical and electronic engineering, Characterization and analytical techniques

## Abstract

The monitoring of vital signs plays a key role in the diagnosis of several diseases. Piezoelectric sensors have been utilized to collect a corresponding representative signal from the chest surface. The subject typically needs to hold his or her breath to eliminate the respiration effect. This work further contributes to the extraction of the corresponding representative vital signs directly from the measured respiration signal. The contraction and expansion of the heart muscles, as well as the respiration activities, will induce a mechanical vibration across the chest wall. The induced vibration is then captured by the piezoelectric sensor placed at the chest surface, which produces an electrical output voltage signal conformally mapped with the respiration-cardiac activities. During breathing, the measured voltage signal is composed of the cardiac cycle activities modulated along with the respiratory cycle activity. A representative model that incorporates the cardiac and respiratory activities is developed and adopted. The piezoelectric and the convolution theories along with Fourier transformation are applied to extract the corresponding cardiac activity signal from the respiration signal. All the results were validated step by step by a conventional apparatus, with good agreement observed.

## Introduction

Monitoring vital signs is essential for daily health and medical diagnosis^1^. Vital signs, such as electrocardiogram (ECG) measurements, heart rate^2^, respiration rate^3^, systolic pressure (SP), and diastolic pressure (DP)^4^, along with pressure pulse play an important role in determining the state of health of a subject^5^. Various methods and systems have recently been developed to monitor such vital signs^6–12^. These vital signs are extracted from the acquired signals that enable more effective safeguarding of health by allowing for the early detection of any disease or abnormality^13–15^. Researchers have recently focused on developing a remote non-contact sensing system having the ability to perform accurate long-term continuous monitoring of human vital signs. Measuring vital signs (such as respiration rates, blood pressure, and heart rates) continuously and remotely without touching the patients can be an invaluable tool for physicians, as it can be used to make life-and-death decisions rapidly and make better decisions when long-term patient data are available^16^. The systems used to monitor vital signs may prevent diseases and enhance quality of life, thus reducing the costs of health care^17^.

Noninvasive vital sign monitoring, which includes measurement of pulse oximetry^18^, capnography, blood pressure (BP) measurement, and the standard five-lead electrocardiogram^19^, has been used for patients who are in the intensive care unit^20^ and in the operating room. A stepped frequency continuous wave (SFCW) radar is also used to measure the heart rate and respiration by transmitting high average power pulses with long duration and narrow bandwidth, then a signal-processing algorithm based on the state space method is applied to extract cardiac and respiration rates from the data measured on a human subject using SFCW radar^21^. Other noninvasive sensors have been implemented in the field. For example, Photo-plethysmography (PPG) sensors, which operate by observing the effect of blood engorgement and composition on light absorption during the systole phase^22^. Furthermore; ECG device that has a capacitive electrode with a shield over conductive foam, and household monitors for heart rate based on ECG techniques using a chest strap^23^.

Piezoelectric transducers incorporating single or multiple arrays have been utilized for the noninvasive monitoring of vital signs^24^. Bifulco *et al*. extracted vital signs by simply filtering the piezoelectric signal. The patient’s ECG signal was simultaneously recorded to provide a time reference of the cardiac activity. The piezoelectric sensor was able to record respiratory movements, seism-cardiogram and heart sounds. These signals can be obtained from the recorded signal by applying simple filters^25^. Klap *et al*. used a piezoelectric sensor to measure respiration and extract ballisto-cardiogram (BCG) waveforms using a proprietary algorithm. The method was not sufficiently robust in extracting the respiration rate because of the difficulty of finding the RR reference value due to environmental noise^26^.

Mizuno *et al*. developed a signal-processing algorithm to extract the essential vital signs from a noisy signal. They focused on monitoring the heart rate of drivers to determine the drowsiness level for safe driving. They adopted seat-embedded piezoelectric sensors to detect the heart rate of the driver. These sensors measure the body vibration caused by the heartbeat, but the signal also contains a large amount of car body vibrations based on the road conditions. To overcome this issue, Mizuno *et al*. proposed a heart rate detection system based on the on-line adaptive filtering technique. The proposed system has a simple structure compared with the offline time-series model (ARX model)-based system and similar performance^27^. Furthermore, the piezoelectric sensors were used in a bed-leaving detection system to monitor the vital signs of the body, such as the respiration rate, blood pressure and human body movements. Piezoelectric sensors were installed inside a pillow and under a mattress or futon. The signal differences between sleeping, awakening, and bed-leaving states were analyzed, leading to the development of a status classification method^28^. Al Ahmad *et al*. proposed the use of a piezoelectric sheet as a contactless cardiac cycle sensor to extract heart rate and blood pressure from the measured output voltage. The employed piezoelectric sheet captures the heart mechanical actions^29^. The generated output electrical voltage is conformally mapped with the heart mechanical activity, as has been presented in another study^30^. The physiological activities of lungs induce mechanical vibrations inside the chest wall^30^. Such vibrations are correlated with the respiration rate^28^. The piezoelectric sheet that is placed on the chest reflects the activities of the lungs and heart into electric signal^31^.

Both piezoelectric and ECG produces a voltage signals corresponded to the electromechanically activities of the heart muscles. Such generated voltage signals are in the range of millimeter of volts. The electrocardiograph require the use of multiple sensor probes to be attached to subject body and have electrical contact. A piezoelectric sensor does not require to have a direct electrical contact with the subject chest. As it has been shown in^29^, a single piezoelectric sensor can extract the ECG signal successfully. The single piezoelectric sensor sheet can detect a multiple vital signs with single shot measurements simultaneously. In addition, the piezoelectric can measure a micro a minute change in the corresponding vital signs.

In this work, novel piezoelectric-based approaches that allow for the extraction of cardiac cycle parameters from a breathing measured signal are investigated. During breathing, the measured voltage signal is composed of the cardiac cyclic activities modulated along with the respiratory cyclic activity. The proposed method utilizes the principles of the piezoelectric and convolution techniques along with Fourier transformation to extract the corresponding signal of the cardiac cycle activities from a breathing signal measured in real time.

## Piezoelectric Working Principle

**Figure 1:**
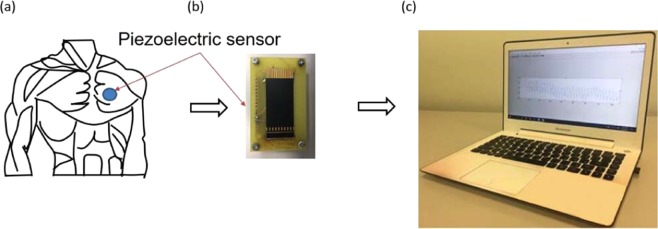
Experimental setup: (**a**) placement of a piezoelectric sheet on the chest, (**b**) piezoelectric bluetooth based system and (**c**) the received data on laptop machine.

Piezoelectric materials exhibit smart properties that make them suitable candidates for use in biomedical devices^32^. The mechanical activities of the heart muscle and lungs induce a vibration inside the chest wall. A piezoelectric cantilever placed on top of the chest surface can collect such vibrations and convert them into an electrical voltage signal^33^. Figure 1(a) illustrates a piezoelectric sheet placed on top of the surface of the human chest.

**Figure 2:**
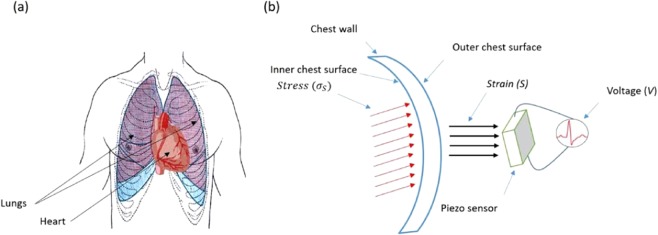
Piezoelectric sensing principle: (**a**) cut-away human body diagram and (**b**) an overview of the coupled mechanical-electrical interactions system model.

Figure 2 illustrates the sensing principle utilizing sheet-type piezoelectric sensor of the cardiac and respiration signals. Figure 2(a) shows a cut-away human body diagram incorporating the heart and lungs organs inside the chest cavity. The effective stress due to the respiration and cardiac activities affecting the surrounding areas of the chest is denoted by σ_s_. This stress is composed of contracting stress (σ_C_) and the relaxation stress (σ_R_) that is generated from mechanical activities of the lungs or and heat. This can be mathematically modelled as per the following equation:

1 $${\sigma }_{{\rm{S}}}={\sigma }_{{\rm{C}}}+{\sigma }_{{\rm{R}}}$$

when a piezoelectric sensor replaced directly on the outer surface of the chest, it will collect the induced vibrations. The sensor output voltage (V(t)) and the induced strain (S(t)) could be expressed as:

2 $$V(t)={c}_{p}{t}_{c}{d}_{31}{\varepsilon }^{-1}S(t)$$

where ε, c_p_, t_c_, and d_31_ are the material dielectric constant, elasticity coefficient, thickness and piezoelectric constant, respectively^29,31^. The induced strain (S(t)) is the available stress at the external chest surface produced by the effective stress (σ_s_) as illustrated in Fig. 2(b). Figure 2(b) proposed a schematic representations for the electro-mechanical interactions due to the respirations and cardiac cycle’s system performances. The heart/lungs mechanical activities through the effective surrounding will induce vibrations in the chest wall, which can be electrically collected using the piezoelectric sensor attached to the external (outer) chest surface as presented by Eq. (2).

In the following sections, the processes used to extract respiration, heart rate and blood pressure values will be discussed sequentially. These parameters are extracted directly from the output voltage of the piezoelectric sheet simultaneously.

## Results and Analysis

This section summarizes the approaches for the extraction of the mutli vital signs parameters from the acquired piezoelectric voltage signal. The piezoelectric transducer incorporated in this work is 46 mm long, 20 mm wide, and 0.26 mm thick, with a lead-zirconate-titanate composition. Table 1 summarizes the important properties; remaining material parameters can be found in the corresponding data sheet^34^. A Bluetooth base piezoelectric system shown in Fig. 1(b) was used to collect the induced vibration on the chest and transmitted wirelessly to a laptop. As shown in Fig. 1(c), the laptop is equipped with MATLAB to receive and plot the output voltage signal. The data is then processed with developed Matlab codes. The use of MATLAB because it is very rich with build-in functions that can help and facilities the extraction of peaks automatically. In MATLAB; the command “findpeaks” was used to find the local maxima (peaks)^35^.

**Table 1:** Properties of the Piezoelectric Sensor.

Piezoelectric Properties	Symbol	Value and Unit
Dielectric constant	ε*T*_11_	4750
Dielectric loss	tan δ	25 × 10^−3^
Conductivity	σ	<1 × 10^−12^ 1/Ωm
Coercive field strength	*E*_*c*_	570 × 10^3^ V/m
Piezoelectric charge Constant	*d*_31_ *d*_33_	315 pm/V 640/V

### Extraction of the respiration rate

**Figure 3:**
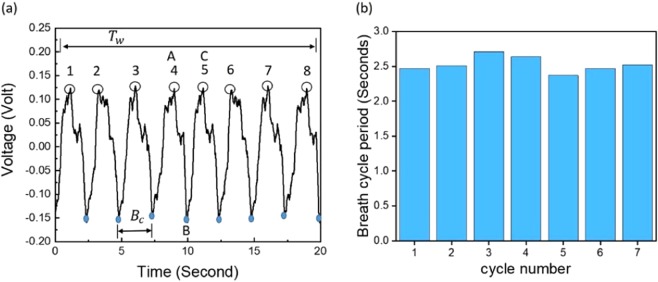
(**a**) Output voltage measured using a piezoelectric sheet while breathing. (**b**) Changes in the breath cycle period over the cycles.

Figure 3(a) displays the measured output voltage time domain signal collected using a piezoelectric sheet placed on top of the chest. The periodic signal has multiple peaks numbered from 1–8 over a 20 s time period. The peaks are conformally mapped to the lungs’ performance. The volume of inlet air to the lungs, which is called the tidal volume, is correlated with the maximum peaks. The slope between the maximum peak (A) and minimum peak (B) reflects the exhalation process, while the slope between the minimum peak (B) and maximum peak (C) reflects the inhalation process of breathing and applies to the remaining peaks.

The peaks of the piezoelectric signal taken upon breathing were defined. The time span between each maximum peak and the next one, as well as between the bottom peaks, represents the breath cycle period (*B*_*c*_). Figure 3(b) illustrates that the cycle period changes slightly from cycle to cycle. By taking the average of the cycles and then calculating the respiration rate per minute by dividing 60 over the time period of the averaged cycle, a respiration rate of 23.7 is obtained for the subject. The following expression was used to extract the respiration rate (breath per minute) from the piezoelectric signal:

3 $$RR={N}^{P}(\frac{60}{{T}_{w}})$$

where *T*_*w*_ is a selected period of time during which the signal is collected and *N*^*P*^ is the number of positive peaks for the selected period of time. The period of breath cycle variation was considered by taking the signal over a selected period and then converting it to breaths per minute. Using Eq. (3) and the data presented in Fig. 3(a), the calculated RR was found to be 24 breaths per minute. In comparison, the measured rate found by counting the breaths taken in one minute was 23 breaths per minute, indicating an error of less than 5%.

### Extraction of the heart rate

**Figure 4:**
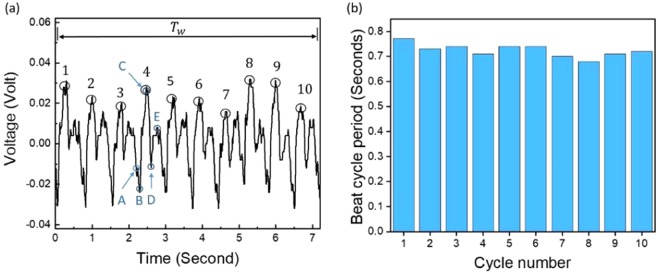
(**a**) Output voltage measured using a piezoelectric sheet while the patient held his or her breath. (**b**) Heartbeat cycle period changes over the cycles.

Heartbeat parameters are extracted from a holding-breath signal since this type of signal is a heart signal without the interference of the respiration effect. The signal was taken over a selected period of time using the same piezoelectric sensor placed on the left side of the chest, as shown in Fig. 4(a). The measured piezoelectric signal is conformally mapped to the heart mechanical activities. There are three main heart actions, which are represented in the output piezoelectric signal in three regions. The first region reflects the atrial contraction and is marked by (A) in Fig. 4(a). This region is generated due to the depolarization of the atrial fibers and aortic valve opening, which causes the contraction.

The second region represents the ventricular contraction and is represented as (B)–(D) in Fig. 4(a). The ventricular contraction is generated due to the depolarization of ventricular fibers. This activation, along with the closing of the aortic valve, makes the ventricular contract. Thus, the stress of the heart is intensified by ventricular depolarization in the ejection or contracting interval, which is between points (B) and (C). The aortic valves are then re-opened, and the repolarization of ventricular fibers is initiated to the relaxation point, represented by (D). Thus, the heart stress decreases until reaching point (D). Repolarizing the ventricular fibers will retrieve its actions and increase the heart stress to reach point (E)^36^.

The peaks of the piezoelectric signal were defined. The time span between the maximum peaks or bottom peaks represents the beat cycle period. The heartbeat cycle period varies slightly from cycle to cycle, as shown in Fig. 4(b). To extract the heart rate from the piezoelectric signal, the beat cycle peaks, denoted by 1–10 in Fig. 4(b), were counted (*N*^*P*^) over a selected period of time (*T*_*w*_), and the following expression was employed:

4 $$HR={N}^{P}(\frac{60}{{T}_{w}})$$

Using Eq. (4) and the data presented in Fig. 4(a), the calculated HR was found to be 83 beats per minute. In comparison, the measured rate found by counting the heartbeat taken in one minute was 87 beats per minute, indicating an error of less than 5%.

### Extraction of the blood pressure parameters

**Figure 5:**
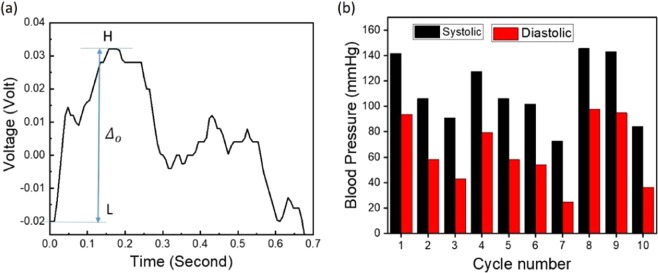
(**a**) A cycle of the signal obtained while the patient was holding his or her breath, labeled with H and L peaks. (**b**) The variations of the SP and DP extracted from each cycle.

Another vital sign was extracted from the piezoelectric signals when the patient was holding his or her breath, namely, the blood pressure. When the signal was collected from the piezoelectric sheet, the systolic pressure (SP) and diastolic pressure (DP) were measured at the same moment conventionally using an electronic sphygmomanometer, or a blood pressure meter. The DP can be extracted from the heartbeat signal by mapping the SP from the conventional meter with the maximum value of the heartbeat signal. First, from the conventional meter readings, we obtain

5 $${\Delta }{P}_{c}=S{P}_{c}-D{P}_{c}$$

where *ΔP*_*c*_ is the difference between the *SP*_*c*_ and *DP*_*c*_ of the conventional meter. On the other hand, the maximum and minimum of the heartbeat signal collected from the piezoelectric signal while holding the breath are represented by H and L, respectively, as shown in Fig. 5(a).

The difference between H and L is *Δ*_*o*_:

6 $${{\Delta }}_{o}=H-L$$

The measured blood pressure values using the conventional meter the piezoelectric signal revealed that each 1 mV in the piezoelectric voltage signal corresponds to 1 mmHg in the conventional meter blood pressure readings. The average value of the maximum peaks (*H*_*av*_) corresponds to the highest reading value in the conventional meter (*SP*_*m*_). The same applies to the cycle level value of the piezoelectric signal (*H*_*cycle*_) and blood pressure value (*SP*_*cycle*_). The following statements relate the piezoelectric signal to the blood pressure values:

7 $${H}_{cycle}=(S{P}_{cycle}{H}_{av})/S{P}_{m}$$

8 $${L}_{cycle}={H}_{cycle}-{{\Delta }}_{v\_cycle}$$

For the entire holding-breath signal, the average *Δ*_*o*_ was found to be 0.0527. The average H was 0.0246, the average L was 0.0281, and *ΔP*_*c*_ was 48. From the previous equations, the SP and DP were extracted from each cycle of the hold-breathing signal, as shown in Fig. 5(b). The average extracted SP was 112, and the average DP was 64. In comparison, the values from the conventional meter for the SP and DP were 112 and 64, respectively.

### Extraction of the hold-breathing signal from the respiration signal

As illustrated previously, the heart rate can be easily extracted from the piezoelectric signal under the hold-breathing situation. However, this extraction method is not practical when asking the patients to continue to hold their breath. Thus, accurately extracting the entire heartbeat signal from the respiration signal becomes critical. The significance of the heartbeat signal involves the ability to extract its multiple vital signs, such as the heart rate, SP and DP. This paper proposes a preprocessing method for removing the respiration-related component from the signal of a piezoelectric sensor located on the chest.

**Figure 6:**
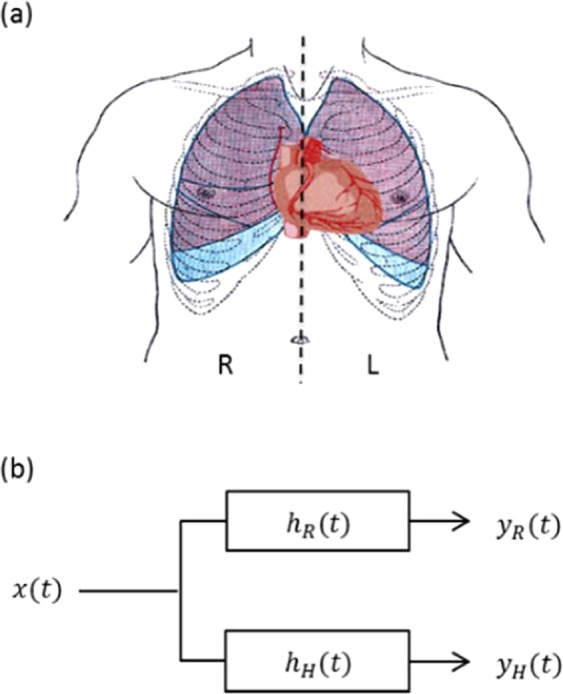
Schematic illustrations: (**a**) human upper body schema (heart, lungs, and thorax); chest is divided into the left side (L) and right side (R). (**b**) System representation and modeling. The excitation signal is represented by *x*(*t*), the respiration signal is represented by *y*_*R*_(*t*) and the heartbeat signal is represented by *y*_*H*_(*t*). The chest wall impulse responses are represented by *h*_*R*_(*t*) for the breathing mode, and *h*_*H*_(*t*) for the hold-breathing mode.

The schematic representation and system modeling of the current approach are illustrated in Fig. 6(a,b), respectively. Figure 6(a) illustrates the left and right sides of the human chest, within which both the lungs and heart are shown.

Based on the causality principle, the presented system model in Fig. 6(b) is proposed to establish the relationships among the different signals. The chest wall exhibits two impulse responses according to whether it is in hold-breathing (*h*_*R*_(*t*) or breathing mode (*h*_*H*_(*t*). Based on this model, one can argue that both breathing and hold-breathing actions are generated from the same excitation signal source. This excitation signal can be represented by a two-level minimum and maximum threshold function. The hold-breathing mode is activated when a minimum threshold is assumed, whereas the breathing mode is activated when its maximum value is assumed.

From the modeling shown in Fig. 6(b) and using convolution theory^37^, *y*_*R*_(*t*) and *y*_*H*_(*t*) are expressed as:

9 $${y}_{R}(t)=x(t)\ast {h}_{R}(t)$$

10 $${y}_{H}(t)=x(t)\ast {h}_{H}(t)$$

where “*” represents the convolution operator. *h*_*R*_(*t*) and *h*_*H*_(*t*) represent the chest wall impulse responses under the breathing and hold-breathing modes, respectively. These responses depend on several parameters, namely, the chest wall thickness and human health conditions. The Fourier transform^38^ of (9) and (10) yields

11 $${Y}_{R}(f)=X(f){H}_{R}(f)$$

12 $${Y}_{H}(f)=X(f){H}_{H}(f)$$

where *Y*_*R*_(*f*), *Y*_*H*_(*f*), *X*(*f*), *H*_*R*_(*f*) and *H*_*H*_(*f*) are the corresponding Fourier transforms of *y*_*R*_(*t*), *y*_*H*_(*t*), *x*(*t*), *h*_*R*_(*t*) and *h*_*H*_(*t*), respectively. The objective is to extract *y*_*H*_(*t*) directly from *y*_*R*_(*t*). Dividing (12) over (11) yields

13 $${Y}_{H}(f)={Y}_{R}(f)(\frac{{H}_{H}(f)}{{H}_{R}(f)})$$

Rearranging (13) and taking the inverse Fourier transform yields

14 $${y}_{H}(t)={F}^{-1}[\frac{{H}_{H}(f)}{{H}_{R}(f)}]\ast {y}_{R}(t)$$

Hence, (14) represents the hold-breathing time domain extracted signal from the measured respiration signal. For further simplification, $\Gamma (t)$ is introduced as follows:

15 $$\Gamma (t)={F}^{-1}[\frac{{H}_{H}(f)}{{H}_{R}(f)}]$$

As long as there are no changes in the physiological status of the subject health in terms of lungs, cardiac diseases and dramatic weight change, $\Gamma (t)$ remains an invariable time domain response. Otherwise, $\Gamma (t)$ will vary accordingly and should be recomputed. Furthermore, since both cardiac and respiratory parameters are expressed per minute, the averaging of the hold-breathing and breathing cycles is introduced. Moreover, to count for the invariable time domain response, the initial measured and averaged cycles for the breathing (${H}_{R0}(f)$) and hold-breathing (${H}_{H0}(f)$) responses are used to compute ${\Gamma }_{0}(t)$ as follows:

16 $${\Gamma }_{0}(t)={F}^{-1}[\frac{{H}_{H0}(f)}{{H}_{R0}(f)}]$$

#### Computation of the chest impulse response

**Figure 7:**
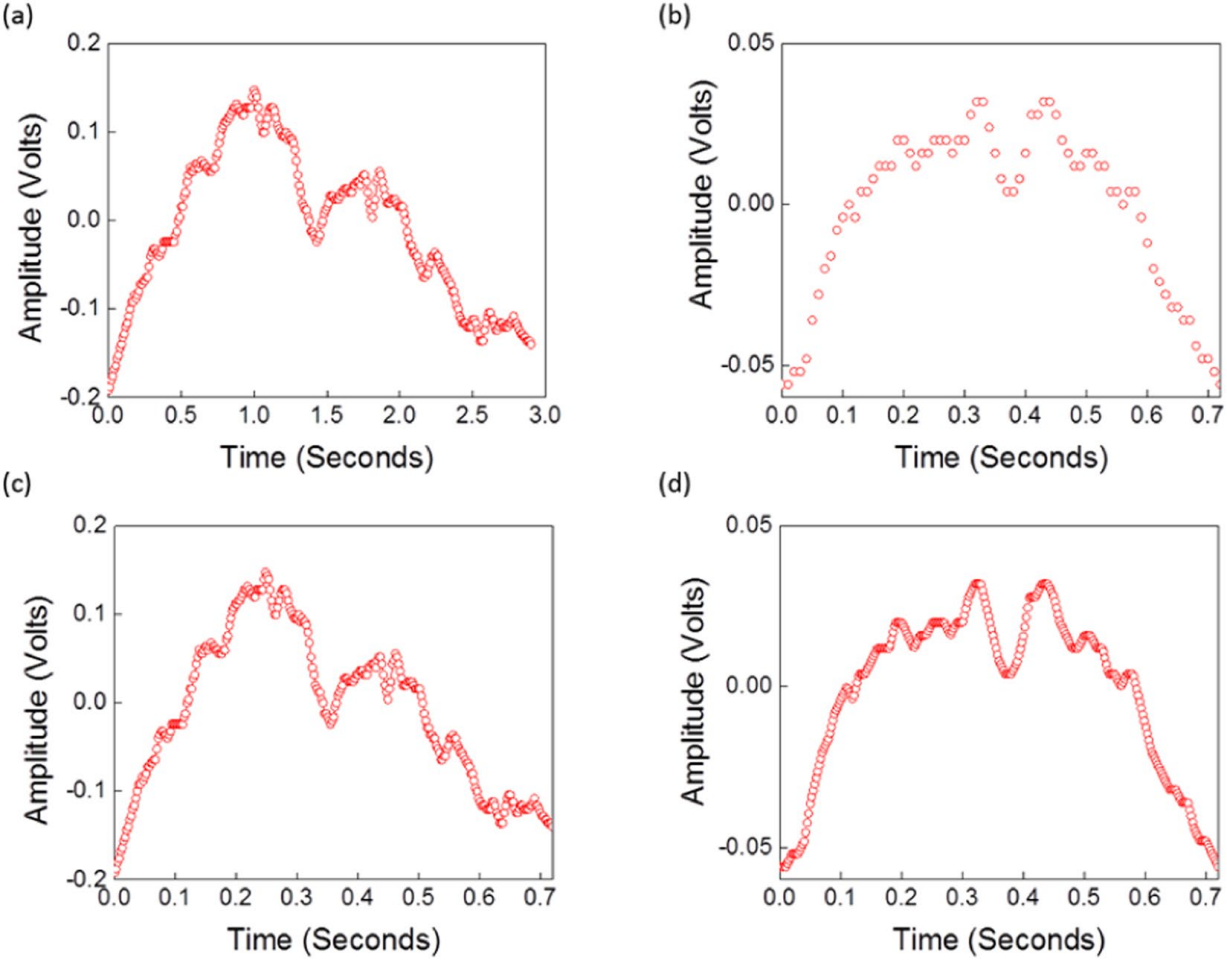
Measured average signals cycles: (**a**) average breathing of three constitute cycles and (**b**) average hold-breathing cycle signals of eight constitute cycles, respectively; (**c**) scaled-down average breathing cycle; (**d**) interpolated average hold-breathing cycle.

To execute the proposed algorithm, because the periodicities of the hold-breathing and breathing cycles are not identical and differ slightly from each other within the same mode, an average cycle for each mode is first computed. Figure 7(a,b) illustrate the average cycle for the breathing and hold-breathing modes, respectively. This averaging is recommended to overcome the slight change in periodicity of the cycles within the same mode. To proceed further, the average breathing cycle has been scaled down in time to have the same period as for the average holding-breath cycle, as depicted in Fig. 7(c). This scaled-down process is important to proceed with the calculations, as both signals should have the same time length. The variability in respiration cycle lengths and heartbeat intervals when calculating the average signal has been considered along with the direct impact of the variation in the respiration time scale on the frequency response scale. Meanwhile, the average cycle of the hold-breathing signal is interpolated to increase the time points, as displayed in Fig. 7(d).

**Figure 8:**
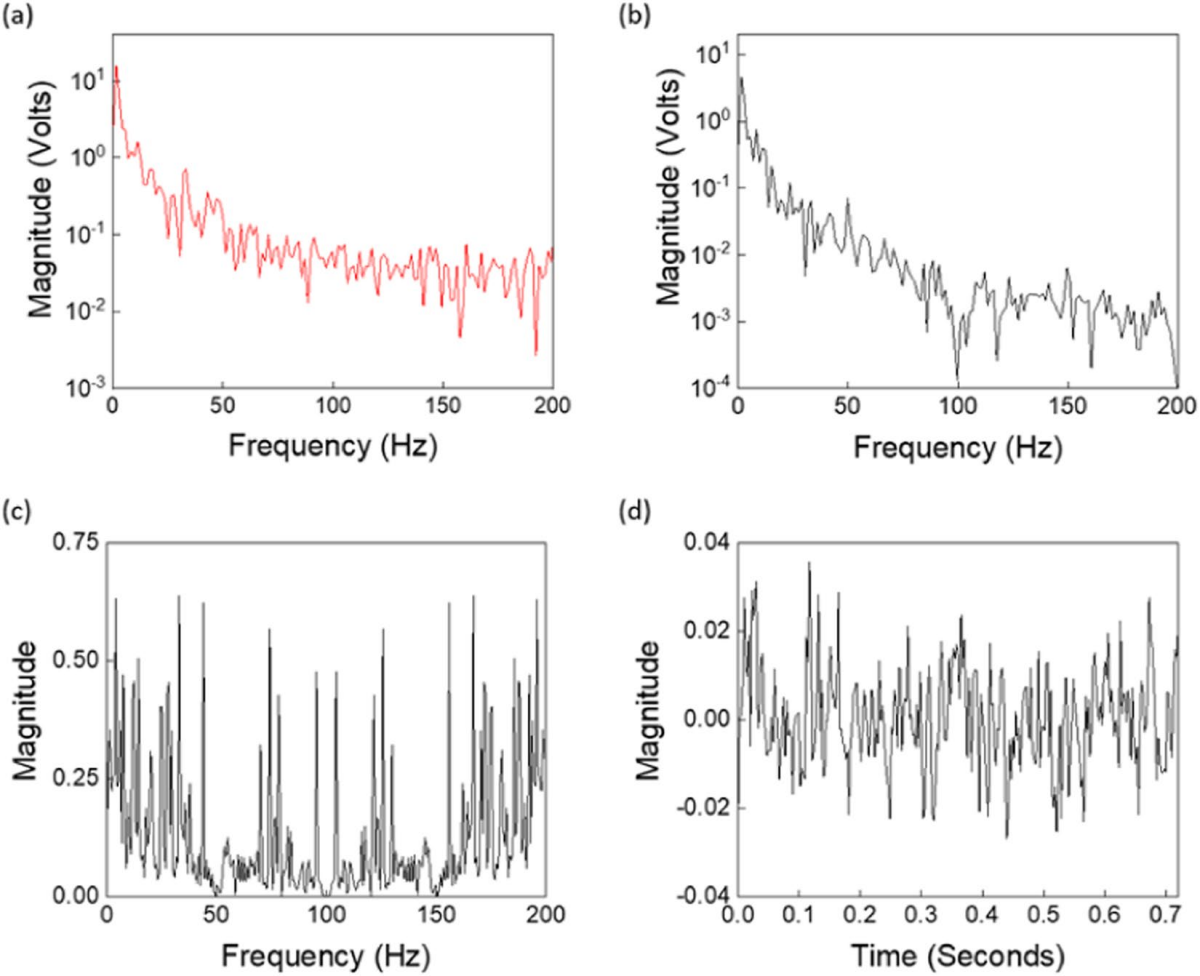
Gamma time and frequency domain signals: (**a**) magnitudes of the frequency domains of the average hold-breathing (Y_H0_(f)) and (**b**) breathing (Y_R0_(f)) cycle signals. (**c**) computed frequency and (**d**) and time domain signals of ${\varGamma }_{0}({\rm{f}})$.

Then, the corresponding frequency domain signals for the average cycles are computed using the Fourier transform^39^. Their magnitudes versus frequency are plotted in Fig. 8(a,b). Recalling Eqs. (14) and (15), the ${\Gamma }_{0}(f)$ function can be computed directly from ${Y}_{H0}(f)$ and ${Y}_{R0}(f)$ as follows:

17 $${\Gamma }_{0}({\rm{f}})={{\rm{Y}}}_{{\rm{H}}0}({\rm{f}})/{{\rm{Y}}}_{{\rm{R}}0}({\rm{f}})$$

where ${Y}_{H0}(f)$ and ${Y}_{R0}(f)$ are the corresponding frequency domain signals for the average hold-breathing and breathing mode cycles, respectively. Figure 8(c,d) represent the frequency and time domain responses of ${\Gamma }_{0}({\rm{f}})$, respectively, which will be utilized in the next subsection to compute the corresponding hold-breathing signal.

#### Hold-breathing signal extraction

To reconstruct the corresponding hold-breathing signal from an instantaneous measured breathing signal, (14) can be reformulated as follows:

18 $${{\rm{y}}}_{{\rm{H}}}({\rm{t}})={{\rm{F}}}^{-1}[(\frac{{{\rm{H}}}_{{\rm{H}}0}({\rm{f}})}{{{\rm{H}}}_{{\rm{R}}0}({\rm{f}})}){{\rm{Y}}}_{{\rm{R}}}({\rm{f}})]$$

Using Eq. (18), an instantaneous cycle of the hold-breathing signal can be reconstructed by multiplying the Fourier transform of an instantaneously measured breathing cycle with the predefined chest impulse response, ${\varGamma }_{0}(f)$, computed in the previous subsection. The instantaneous hold-breathing cycle (${y}_{H}(t)$) can then be extracted by finding the inverse Fourier transform of this multiplication.

### Validation of the presented approaches

For further demonstration of the concept and its validation; the breath and hold-breathing signals were collected from different human subjects using the Bluetooth-enabled piezoelectric system. In addition to that, the six subjects corresponding vital signs: respiration rate, heartbeat rate, and pressure pulse were measured using the conventional equipment. Table 2 summarizes the measured vital signs using conventional equipment and methods along with weight, height, age and subject’s gender. The respiration rate was measured using Zephyr from Medtronic/US^40^. The heart beat and blood pressures were measured using the BP742N Blood Pressure monitor from Omron healthcare^39^.

**Table 2:** Measured parameters for using conventional meters*.

Case	W (kg)	H (meter)	RR (Breath/min)	PP (mmHg)	HB (Beats/min)	Age (Year)	Gender
1	55	1.61	21	35	74	26	F
2	40	1.53	26	32	83	28	F
3	51	1.62	18	29	65	29	F
4	50	1.54	27	32	70	33	F
5	58	1.59	23	31	79	27	F
6	53	1.62	21	38	60	25	M
8	70	1.50	25	29	95	22	M
9	60	1.65	20	24	77	25	M
10	65	1.63	24	25	63	23	M

*Where; W: weigh in kg; H: height in meter; RR: respiration rate in breath per minute; PP: pulse pressure in mmHg; HB: heartbeat in beat per minute.

**Figure 9:**
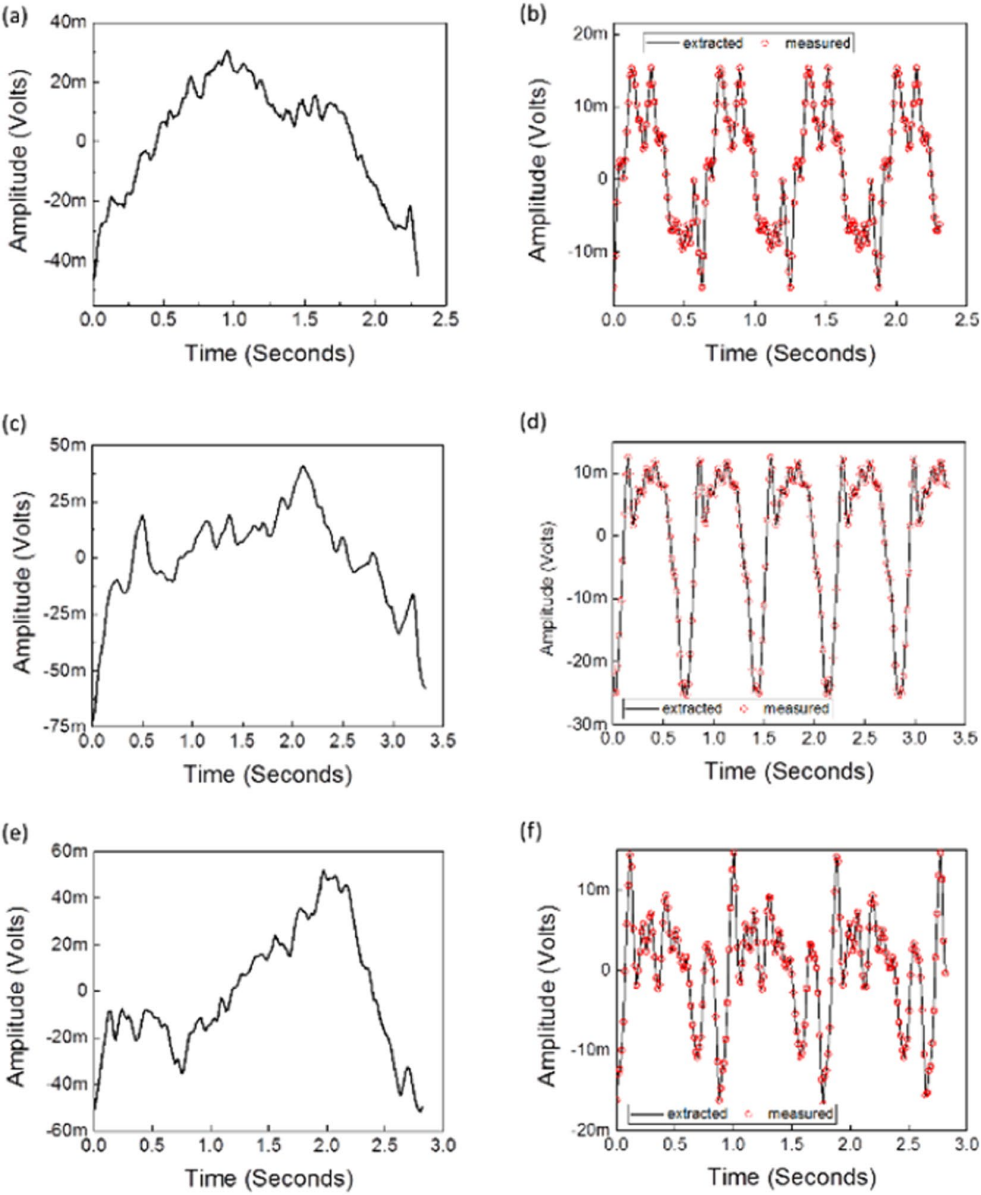
Selected cases (8, 9 and 10, as per Tables 2 and 3): (**a**,**c**,**e**) are the instantaneously measured breathing cycles, and (**b**,**d**,**f**) are the corresponding constructed hold-breathing cycles superimposed with the average measured hold-breathing cycles. (**a**,**b**) corresponds to case #8, (**c**,**d**) corresponds to case #9 and (**e**,**f**) corresponds to case #10.

Figure 9 shows the average respiration cycles along with the corresponding hold-breathing signal superimposed with the averaged measured hold-breathing cycles of selected cases (8, 9 and 10, as per Tables 2 and 3). Figure 9(a,c,e) are the instantaneously measured breathing cycles, and (b), (d) and (f) are their corresponding extracted hold-breathing corresponding cycles superimposed with the average measured hold-breathing cycles.

**Table 3:** Extracted parameters from reconstructed Hold breathing cycles*.

Case	Estimated			Error (%)		
	RR	HB	PP	ERR	EHB	EPP
1	24	66	27	14	10	22
2	26	75	29	0	10	9
4	20	75	32	11	15	10
5	26	66	21	4	6	34
6	30	80	30	30	1	3
7	20	60	27	5	0	29
8	26	100	27	4	5	7
9	18	75	22	10	3	8
10	22	65	27	8	3	8
Average error ± STD (%)				10 ± 6	6 ± 3.5	15 ± 7.5

*ERR, EHB and EPP are the errors in RR rate, HB rate and PP, respectively.

Now, the corresponding RHB and PP parameters have been estimated from the signal represented in Fig. 9. The current approach has been successfully employed to extract the hold breathing signals. Figure 9(b,d,f) revealed that measured and extracted are well cooperated with each other. The error in regeneration of the holding-breath signal is very negligible. With the algorithms presented in ref. ^19^; the corresponding computed parameters are summarized in Table 3.

The hold-breathing cycles extracted from the relevant respiration signals are identical to the measured hold-breathing cycles. Hence, the error of this extraction process is almost negligible, which proves the validity of the algorithm.

The exact location of the sensor affects the transfer functions, and the appropriate position has been optimized experimentally. The left side of the chest is the optimal position for the piezoelectric sheet to extract the hold-breathing signal. Nevertheless, the right side of the developed algorithm can extract the hold-breathing signal from the piezoelectric sheet placed on the right side of the chest, which is beneficial for patients with special conditions.

The differences in the respiration and hold-breathing patterns could be due to the differences in chest thicknesses of the different subjects under study. The chest thickness will cause delays and compression of the signal. Also these differences can be correlated to the lungs organ and heart muscle themselves. It is mostly unlikely that all human beings will have same heart and lungs characteristics in terms of size, shape; walls thicknesses and pumping capabilities, etc. Moreover their physiology will absolutely differ from person to another person.

## Discussions

The following strategy has been adopted in the determination of the maximum and minimum thresholds to activate the hold-breathing or breathing modes. Indeed the two modes can be easily identified by observing the signal cycle period and its corresponding magnitude. As the typical heartbeat ranges between 60–100 beats/min: if the measured cycle period is equal to 0.75 ± 0.25 seconds, this clearly represents the hold breathing signal (i.e. cardiac signal). As the typical respiration breath rate ranges between 12–25 breaths/min for adults. If the measured cycle period is equal to be 3.25 ± 0.35 seconds, this clearly represents the breathing signal (i.e. respiration signal). Furthermore, it is worth to mention that both the shape profile and the cycle period of the respiration and cardiac signals do not change. The signal amplitude depending on the material piezoelectric coefficient incorporated in the sensor. Both respiration and beating rates are independent on the employed piezoelectric material coefficients and characteristics. Also the minor feature in the shape profile of the output signal of the PZT sensor does depend on the sensitivity of the material, i.e. the piezoelectric coefficients. Incorporated materials with higher coefficients pronounce more fine features. Nevertheless, the amplitude of the signal strongly depend on the piezoelectric coefficients. The higher the coefficient is, yields more signal strength. Therefore, the cycle period is the explicit parameter that can be used as a threshold to decide whether it is a respiration or hold-breathing mode. Hence; if the cycle period is less than 1.25 to be hold breathing and if more than that to be breathing mode.

The breathing signal is three times larger in amplitude than the hold-breathing signal, the produced electrical voltage corresponds to only cardiac cycle contraction and expansion activities. Meanwhile, during the breathing mode, the generated electrical voltage is due to both heart muscle and lung activities simultaneously. The hold-breathing signal exhibits a higher frequency than the breathing signal. The frequency of the latter signal represents the respiration rate, whereas the frequency of the former signal represents the heart rate. The heart rate is always higher than the respiration rate because the lungs normally expands and contracts up to 20 times a minute to supply oxygen to be distributed throughout the body and expel carbon dioxide that has been created throughout the body^41^. Meanwhile, the heart muscle expands and contracts up to 100 times a minute to supply blood to the entire body^37,42^.

The sensitivity of the detection of the maximum and minimum peaks in the output signals of the piezoelectric depends on the piezoelectric voltage coefficient and the thickness of the incorporated material. For the employed current sensor, the positive peak is considered to be above 0.75 volt and the negative peak to be below −0.75 Volt for the breathing mode. Meanwhile for the hold breathing mode, the positive peak is considered to be above 0.015 volt and the negative peak to be greater than −0.015 in magnitude. The cycle period has been determined for the respiration by the number of two zero crossing within which a maximum peak occurs. The respiration period then starts from the minimum point before the first zero crossing and ends with the second adjacent minimum point after the second crossing. Meanwhile the cycle for hold-breathing has been determined by the number of four zero crossing within which a maximum peak occurred. The hold breathing period then starts from the minimum point before the first zero crossing and ends with the last adjacent minimum point after the second crossing.

To avoid the miss-detection or over-detection due to the variance of the peak amplitude, PZT material has been used in fabricating the sensor as it is known to be the most sensitive piezoelectric material. Furthermore, during processing the zero crossings have been used to define the cycles and by that detect the peaks accurately.

To overcome the small noises of talking, or speaking during the measurements, vital signs extraction process have been perform after an averaging process. The averaging cycle was found over a specific time. There is not any constrain on the number of cycles that could be taken for the averaging, but for consistency all the measurements were taken for the same number of cycles.

The respiration and heartbeat signals are affected by emotional status of the subject, these small fluctuations are eliminated by the averaging process. The averaging process can affect the shape of the signal butt the final result in terms of heartbeat and respiration will not exceed an error of one cycle per minute. On the other hand, detecting abnormalities might be not accurate due to that. More studying should be performed for the future to be able to detect abnormalities affectively.

Moreover; the error is defined to be the [(conventional − measured)/conventional]. It could be due to the utilized mechanism principles of operation or even due to the averaging process of the signal and due to the possible delay of the material response of the heart activity from the chest membrane and material properties. The piezoelectric material exhibits high dielectric constant which results in parallel plate capacitance value. Placing the sensor in different areas on the chest has been studied to find the optimal position to place the sensor. It was found that the optimal position is on the left side of the chest above the heart. Nevertheless, any other position of the abdomen area gave a good results as the developed algorithm can work well if the sensor placed at any part of the abdomen area.

The main application of the current work is the monitoring of multiple vital signs of patients, such as the respiration rate from the piezoelectric signal, heart rate, and blood pressure from the extracted heartbeat signal. The advantage of the algorithm is that it does not require the patient to hold his or her breath while measuring and is improved compared to the existing algorithm, the proposed algorithm is able to measure a patient’s heart rate and blood pressure using one small piezoelectric sheet while moving normally. Being able to measure multiple vital signs from one piezoelectric sheet makes the monitoring easier and more comfortable for patients, with fewer wiring complications. Moreover, the proposed method can extract the heart rate signal from the right side of the chest for special cases where the patient cannot tolerate electric monitors on top of the heart position.

Sullivan *et al*.^43^ proposed a system composed of piezoelectric sensors and signal conditioning circuits and signal processing to extract multiple vital signs. They used sensors attached to the body of a person to detect signals, including mechanical, thermal and acoustic signals reflecting cardiac output, cardiac function, internal bleeding, respiration, pulse, apnea, and temperature. For noise reduction, they have used a separate piezoelectric sensor that was not attached to the person was used. Instead, the sensor was exposed to the environmental acoustic and vibration signals, while the sensor attached to the body was exposed to the environment as well as the body signals. Subtraction of one input from the other allows for noise reduction and yields only the signal of the body using initial measurements as control signals. They described that some of those measurements were related to heart rate measurements in the absence of respiration. The signal was run through filters and other signal-processing algorithms. The signal-processing techniques utilized prior knowledge of the expected signals to extract the desired information from the piezoelectric signal. The resultant signal was analyzed through routine techniques, including fast Fourier transform, to identify a primary signal frequency representing respiration and a second signal frequency representing heart rate. However, the signal was acquired with the subject being still and speechless during the data acquisition process.

The difference between the work presented in this paper and the work of Sullivan *et al*. is that Sullivan *et al*. defined the respiration signal to include only actions relating to respiratory activity. They used signal cancellation to reduce noise in the measured signal. Their work required the patient to hold his or her breath while extracting the heart rate parameters. Moreover, they simply referred to the application of “digital filters and known processing techniques” to achieve the separation of the signals.

The current work represents a paradigm shift for estimating the hold-breathing signal from the respiration signal. The experimental outcomes reveal the power of the proposed technique in terms of the high similarity between the measured and constructed signals. This approach will facilitate the development of new sensors to detect and identify respiration and heart abnormalities. Hence, this algorithm proved its ability to extract heart rate signals utilizing piezoelectric sensors placed away from the heart position, which is beneficial for patients with special conditions. Furthermore, the piezoelectric based sensor can be implemented in a compact wireless system that can transmitter the data over distance without the need to connect the subject to any equipment preventing him from movement. The piezoelectric sensor does not require any electrical contact with the subject as in the ECG electrode probing system. The piezoelectric output incorporates fine features that can be used for simultaneous multiple vital sign determination as well abnormalities identifications.

One of the limitations of the algorithm is that the piezoelectric sheet should be placed on the chest and abdomen area. Furthermore, the algorithm can handle small noises, such as speaking, eating, and small movements, while measuring; these noises can be removed from the averaging process. The algorithm must be further improved to neglect the effect of major noises that could occur while moving.

The proposed approaches for the extraction of multiple vital signs simultaneously can be utilized in several applications. With further development and optimization it can be used in the detection of heart failure, lungs and cardiac abnormalities detection^44^. Currently there are huge interests in developing e-doctor based system that incorporates several defined medical conditions composed of several vital signs^45^. The physiological multi vital signs based emotion recognition system combined with statistical analysis, the system was able to recognize emotion on three basic emotional states^46^. It also can be employed in developing an automatic productive assessment systems to assess the ability of capturing the effect of construction workers’ happiness on their productivity using physiological signals^47^.

## Conclusion

This study proposed a novel piezoelectric-based technique to extract the hold-breathing signal from the breathing signal utilizing piezoelectric theory and signal-processing techniques. The piezoelectric-based transducer, which is placed on the subject’s chest, is used to collect the breathing signals. The piezoelectric transducer provides noninvasive and contactless connections. With the current approach, the patient is no longer required to hold his or her breath to collect the cardiac signals, as is required by other methods. The study outcomes revealed that the developed technique is simple, reliable, and easy to handle. Therefore, it causes minimal inconvenience to clinics and patients, particularly heart patients with special conditions.

## Ethics Statement

- The authors confirm that all methods were carried out in accordance with relevant guidelines and regulations.
- The authors confirm that all experimental protocols were approved by *Al Ain Medical District Human Research Ethics Committee* – Protocol N0. 14/67- cardiac and inspiratory piezoelectric study.
- The authors are confirming that informed consent was obtained from all subjects.
